# Lectures on the Surgical Disorders of the Urinary Organs

**Published:** 1894-06

**Authors:** 


*Lectures on the Surgical Disorders of the Urinary Organs.* By Reginald Harrison, F.R.C.S. Fourth Edition. Pp. xiv., 588. London: J. & A. Churchill. 1893.

The subject-matter included in this volume originally formed portions of successive courses of lectures delivered at the Liverpool Royal Infirmary, together with lectures delivered before the Medical Society of London and the Royal College of Surgeons. The whole forms a somewhat ponderous volume, in which the lecture form has been maintained throughout—a method which is somewhat tedious to the reader, although it may possess some compensatory advantages for the writer. Mr. Reginald Harrison is so well known as an authority on all matters relating to the surgery of the urinary organs, that it is unnecessary for us to call attention to the excellence of the matter contained in these lectures. They are full of good practical suggestions, illustrated by cases from the author's experience, and will amply repay a careful perusal. The illustrations are largely taken from the instrument-maker's catalogue; but there are some good original drawings and a few very bad ones, among which we would especially notice one on page 160. We are somewhat surprised at the very faint praise with which Mr. Harrison speaks of the operation of litholapaxy in children. He still clings to the old operation of lateral lithotomy, which we suppose is only natural in one who has practised it so successfully. We shall have to wait until a new generation of surgeons has risen up in this country before this grand old *tour de maître* is finally condemned to the limbo of forgotten methods of operating.

